# An MEF-Based Localization Algorithm against Outliers in Wireless Sensor Networks

**DOI:** 10.3390/s16071041

**Published:** 2016-07-07

**Authors:** Dandan Wang, Jiangwen Wan, Meimei Wang, Qiang Zhang

**Affiliations:** School of Instrumentation Science and Opto-electronics Engineering, Beihang University, Xueyuan Road No.37, Haidian District, Beijing 100191, China; wangdd@buaa.edu.cn (D.W.); may@aspe.buaa.edu.cn (M.W.); youleyuanzq@buaa.edu.cn (Q.Z.)

**Keywords:** wireless sensor networks, localization, outliers, maximum entropy principle, fuzzy set theory

## Abstract

Precise localization has attracted considerable interest in Wireless Sensor Networks (WSNs) localization systems. Due to the internal or external disturbance, the existence of the outliers, including both the distance outliers and the anchor outliers, severely decreases the localization accuracy. In order to eliminate both kinds of outliers simultaneously, an outlier detection method is proposed based on the maximum entropy principle and fuzzy set theory. Since not all the outliers can be detected in the detection process, the Maximum Entropy Function (MEF) method is utilized to tolerate the errors and calculate the optimal estimated locations of unknown nodes. Simulation results demonstrate that the proposed localization method remains stable while the outliers vary. Moreover, the localization accuracy is highly improved by wisely rejecting outliers.

## 1. Introduction

Wireless Sensor Networks (WSNs), the networks of sensor nodes, have been widely used in many promising applications such as condition monitoring, target tracking, and home security. Precise localization plays an important role in WSNs localization systems. From the viewpoint of localization systems, there are two types of sensor nodes in WSNs. Anchor nodes, also known as beacon nodes, can obtain their location directly by using manual placement or Global Positioning System (GPS); unknown nodes, also known as regular nodes, derive their locations through localization methods. Up to now, most existing localization algorithms of WSNs could be classified as either range-based localization [1,2] or range-free localization [3,4]. Range-based localization algorithms use absolute point-to-point range measurements (distance or angle) to estimate unknown nodes’ locations, while range-free localization algorithms depend on the contents of received messages. In this paper, the range-based localization methods are taken into consideration, since they are normally of high localization accuracy [5].

In localization methods, the calculation of unknown node’s positions heavily relies on primary data, which are the distances between neighboring nodes and the position knowledge of anchors. In many applications of WSNs, sensor nodes are vulnerable to the internal or external disturbance. As a result of the inference, the measured distances and anchor positions can deviate from their true values. These inaccurate values are called outliers, including both the distance outliers and the anchor outliers. Due to the existence of outliers, the usage of such corrupted data can severely degrade the localization accuracy. Hence, the outlier detection process [6] is a necessary step to assure data quality in localization process. Up to the present, most existing outlier detection methods [7,8,9] simply assume that either distance or anchor position is the outlier, thus they are not comprehensive detecting methods. Furthermore, when the difference between the outlier value and the normal value is small enough, the outlier will not be detected, thus these methods will clearly be invalid. Therefore, an error-tolerant localization method is greatly needed to calculate the estimated locations of unknown nodes in the presence of undetected outliers. The error-tolerant localization method is a positioning refinement process which allows the existence of undetected outliers instead of discarding them. Through the error-tolerant localization process, the accuracy of localization will be improved efficiently.

In this paper, a novel secure localization method is developed to reject both the distance outliers and anchor outliers. Firstly, the uncertain value of the measured distances is obtained based on the maximum entropy theory in the lack of ranging error distribution. The uncertain value is served as the threshold in the membership function, which is compared with the difference between the Euclidean distance and the measured distance between every two neighboring anchor nodes. The Euclidean distance is calculated by the coordinates of the two anchors while the measured distance is obtained by the range-based methods. Secondly, a trust evaluation model is constructed based on the fuzzy set theory. In the trust evaluation model, a membership function is used to calculate the mutual trust values of anchor nodes. Through the data fusion, the trust value of each anchor node is obtained, and the lower trust value nodes are discarded. Finally, the Maximum Entropy Function (MEF) method is used to calculate the optimal estimated locations of unknown nodes by using the trustable data. Simulations demonstrate that the outliers can be detected effectively and the localization method can achieve high accuracy.

The rest of this paper is organized as follows. Section 2 reviews the related works. Section 3 shows a preliminary structure of sensor nodes localization system. Section 4 describes the outlier detection method. Section 5 presents the MEF method for calculating the optimal estimated locations of unknown nodes. Section 6 shows the simulation results. Finally, Section 7 concludes this paper.

## 2. Related Works

Generally speaking, the outliers have three anomalous causes: (1) hardware malfunctions; (2) environment interferences; or (3) malicious attacks [10]. For instance, in Time of Arrival (ToA) and Time Difference of Arrival (TDoA) systems, the transmission time or reception time of a packet can be delayed, thus resulting in distance enlargement or distance reduction [11]. In Received Signal Strength Indicator (RSSI)-based localization systems, the signal strength may be unstable or shadowed in the presence of natural or artificial interferences [12]. In malicious attacks, an attacker can increase or decrease the transmission power to make the measured distances deviate from their true values. In addition, the attacker can also capture anchor nodes to declare fake anchor positions to generate anchor outliers [13].

In practice, the existence of outliers is a fact that cannot be neglected for localization algorithms. In the case of distance outliers, a consistency check method [14] has been proposed to filter out the malicious beacon signals. The signals contain measured distance outliers on the basis of the “consistency”. However, if the attackers do not revise the measured distances randomly, but make the modified distances be consistent, the strategy mentioned above will be failed under this scenario. In literature [15], linear equations are used to describe the localization problem. Hence, the norm and linear programming are applied to detect the outliers and avoid the wild measurements in the final solution. To deal with noisy and outlier ranging results, a theoretical foundation [16] has been built to identify distance outliers based on graph embeddability and rigidity theory. However, rigidity theory requires high ranging accuracy and it is computationally intensive. By applying the rigidity theory, the concept of verifiable edges [17] has been presented and the conditions for an edge to be verifiable have been derived. On this basis, the paper designs outlier detection method which explicitly eliminates ranges with large errors. However, facing with the undetected small outliers, the method would lose efficacy. In summary, based on the detection target, the literatures [14,15,16,17] mentioned above ignore the influence of anchor outliers. Therefore, these methods are one-sided.

Regarding anchor outliers, a scheme named Localization Anomaly Detection (LAD) [18] is put forward to detect malicious anchor node. The scheme attempts to perform compromise resistant localization without removing the malicious anchors. To monitor and timely detect anchor outliers in large-scale WSNs, a rule-based anomaly detection system, called RADS [19], has been proposed. In conclusion, the results of the algorithms [18,19] which are committed to eliminate anchor outliers are not comprehensive without analyzing the influence of ranges with large errors.

With respect to both outliers, an innovative modular solution [20], featuring two lightweight modules, has been developed. One is attack detection module that harnesses simple geometric triangular rules and an efficient voting technique. The other is secure localization module that computes and clusters certain reference points to estimate the coordinate of the unknown nodes. In [21], a novel algorithm, called neighbor constraint assisted distributed localization (NCA-DL), has been proposed. The method introduces the geometric constraints to detect outliers. To make localization attack-tolerant, a robust statistical method [22] has been presented. By using an adaptive least squares and Least Median Squares (LMS) position estimator, the method is capable of switching to a robust mode when the outliers exist. As a summary of the foregoing, the methods [20,21,22] could dispel either the anchor outliers or distance outliers. If two kinds of outliers both exit, the geometric constraints and statistical method will become invalid to filter out malicious colluding beacons or the beacon whose measured distance and coordinates change at the same time. In addition, to reduce the impact of both outliers simultaneously, Jin et al. [23] has put forward a trilateral localization algorithm for outliers suppression. However, the study of the paper focuses on the error of the algorithm itself, and discusses the stability of equations. This outliers excluded are just a portion of malicious beacons. Reference [24] designs a Beta Reputation System-based Localization (BRSL) algorithm to mainly detect and eliminate both outliers, but the Taylor-series least squares localization algorithm utilized after trust evaluation phase can’t reach high accuracy.

## 3. Preliminaries

**Figure 1:**
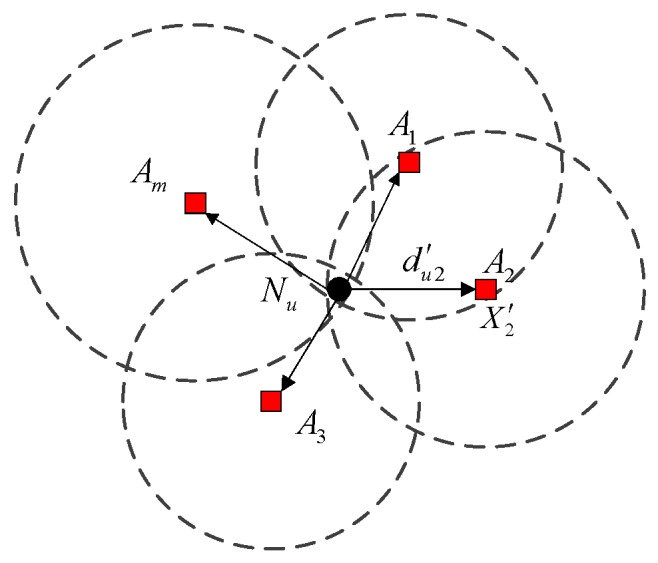
The measured distance $d_{u2}^{\prime }$ and declared anchor position $X_{2}^{\prime }$ is normal.

A WSN consists of two types of nodes, namely anchor nodes and unknown nodes. The anchor nodes are specially equipped and aware of their coordinates after deployment. The unknown nodes, whose positions are yet to be discovered, estimate their locations by measuring distances to neighboring anchor nodes. All the nodes are randomly deployed in a 2D spatial region. The communication radius of unknown or anchor nodes is R. Every node is capable of measuring the distance to any of its immediate neighbors through measurement techniques such as RSS, ToA or TDoA. As shown in Figure 1, when the unknown node $N_{u}$ gets enough measured distances $d_{ui}^{\prime }$ to anchor nodes $N_{i}\left(i=1,2,\ldots ,m\right)$, m≥3, a system of Euclidean equations can be set up according to the trilateration:

(1) $$\{\begin{array}{c} {\left(x_{1}-x_{u}\right)}^{2}+{\left(y_{1}-y_{u}\right)}^{2}=d_{u1}^{\prime 2}\\ {\left(x_{2}-x_{u}\right)}^{2}+{\left(y_{2}-y_{u}\right)}^{2}=d_{u2}^{\prime 2}\\ \vdots \\ {\left(x_{m}-x_{u}\right)}^{2}+{\left(y_{m}-y_{u}\right)}^{2}=d_{um}^{\prime 2} \end{array}$$

where $X_{u}={\left[x_{u},y_{u}\right]}^{T}$ is $N_{u}$’s coordinates that need to be estimated, $X_{i}={\left[x_{i},y_{i}\right]}^{T}$ is anchor node $N_{i}$’s declared position, and $d_{ui}^{\prime }$ is the measured distance between the anchor node and the unknown node.

Generally, $X_{u}$ should be located in the intersection of m circles, of which the centers and radiuses are $X_{i}$ and $d_{ui}^{\prime }$, respectively. The smaller the intersection is, the more accurately $X_{u}$ can be pinpointed. When both $X_{u}$ and $d_{ui}^{\prime }$ are accurate, the $X_{u}$ can be well estimated by solving Equation (1). However, if the distance outliers or the anchor outliers exist, the system would incorrectly estimate the $X_{u}$ to a location that deviate far from its physical position.

**Figure 2:**
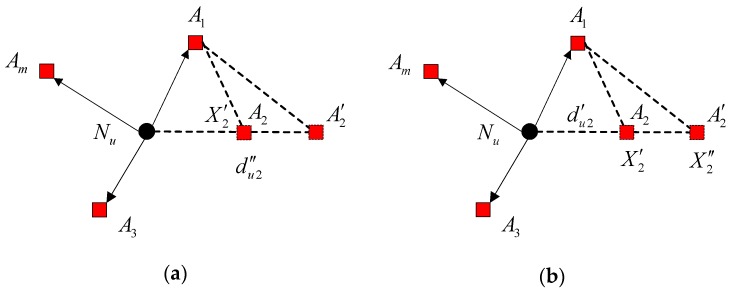
(**a**) The distance-measuring process is disturbed; (**b**) the declared position of the anchor node is inaccurate.

The measured distance can be expressed as $d_{ui}^{\prime }=d_{ui}+e_{r}$, and the declared anchor position can be expressed as $X_{i}^{\prime }=\left[x_{i}+e_{px},y_{i}+e_{py}\right]$, where $e_{r}$ and $e_{p}$ are the ranging error and the anchor position error, respectively. Ranging error $e_{r}$ is the difference between real distance and measured distance between two sensor nodes. Position error $e_{p}$ is the difference between the real position of the anchor node and the received position of the anchor node. If the distance-measuring process is disturbed, the measured distance $d_{u2}^{\prime }$ between anchor node $A_{2}$ and unknown node$N_{u}$, as well as the measured distance between anchor node $A_{2}$ and $A_{1}$, will be enlarged or reduced. Take the enlarged case for example. As shown in Figure 2a, the distance outlier is $d_{u2}^{\prime \prime }=d_{u2}+e_{r}+d_{a}=d_{u2}^{\prime }+d_{a}$, where $d_{a}$ is the enlarged distance, and $d_{u2}$ is the real distance between anchor node $A_{2}$ and unknown node $N_{u}$. Meanwhile, the Euclidean distance between $A_{1}$ and $A_{2}$, i.e., $\left\|A_{1}A_{2}\right\|$, is different from their measured distance $\left\|A_{1}A_{2}^{\prime }\right\|$. In addition, if the measured distance $d_{u2}^{\prime }$, as well as the measured distance between anchor node $A_{2}$ and $A_{1}$, is reduced, the computed distance between $A_{1}$ and $A_{2}$, i.e. $\left\|A_{1}A_{2}\right\|$, is also different from its measured distance $\left\|A_{1}A_{2}^{\prime }\right\|$. As shown in Figure 2b, if anchor node $A_{2}$ is malicious, the declared anchor position may deviate far from the true position. The anchor outlier is defined as $X_{2}^{\prime \prime }=\left[x_{2}+e_{px}+d_{x},y_{2}+e_{py}+d_{y}\right]$, where $\left[d_{x},d_{y}\right]$ is the offset distance. In addition, the Euclidean distance between $A_{1}$ and $A_{2}$, i.e. $\left\|A_{1}A_{2}^{\prime }\right\|$, is not equal to their measurement distance $\left\|A_{1}A_{2}\right\|$. Based on the above discussion, these outliers will severely degrade the localization accuracy. Therefore, it is necessary to eliminate the outliers in localization systems.

The measured distance and anchor positions exist in pairs in the localization systems. Considering two neighboring anchor nodes around an unknown node, no matter the measured distance between the two anchor nodes is enlarged or the declared position of one anchor node deviates far from the true position, the Euclidean distance will be different from the measured distance between the two anchor nodes. Therefore, no matter the distance outliers or the anchor outliers exist, or how they are generated, the difference between the Euclidean distance and the measured distance, as well as the cooperation of anchor nodes, can be utilized to detect the outliers.

## 4. Outlier Detection Method

In this section, firstly, the uncertain value of measured distances is calculated based on maximum entropy theory by using the ranging error priori information. Then a trust evaluation model is constructed based on the fuzzy set theory by using the uncertain value and the difference between the Euclidean distance and the measured distance. In the trust evaluation model, the trust value of each anchor node can be obtained.

### 4.1. Calculation of the Entropy Uncertainty

Based on the maximum entropy theory, the uncertain value of the measured distance can be obtained by utilizing the mean and standard deviation of ranging error in this section. The information entropy $H\left(e_{r}\right)$ [25] of ranging error can be calculated as Formula (2),

(2) $$H\left(e_{r}\right)=-\sum_{i=1}^{n}p\left(e_{ri}\right)\text{ln}\;p\left(e_{ri}\right)$$

where $p\left(e_{r}\right)$ is the probability density function of ranging error. 

The ranging error $e_{r}$ is assumed to appear in $\left[e_{r1},e_{r2}\right]$ with equal probability before measuring. Then, after measurement, the estimated ranging error $e_{r}^{\prime }$ with bias ±U is obtained, where U is the entropy uncertainty of ranging error. Hence the true value of ranging error appears in $\left[e_{r}^{\prime }-U,e_{r}^{\prime }+U\right]$. The information entropy of $e_{r}^{\prime }$ can be calculated as Formula (3).

(3) $$H\left(e_{r}^{\prime }\right)=-\int_{e_{r}^{\prime }-U}^{e_{r}^{\prime }+U}\frac{1}{2U}\text{ln}\frac{1}{2U}de_{r}=\text{ln}\;2U$$

As we all know, the probability density function of Gaussian distribution $N\left(0,\sigma^{2}\right)$ is

(4) $$p\left(e_{r}\right)=\frac{1}{\sqrt{2\pi }\sigma }\text{exp}\left(-\frac{{e_{r}}^{2}}{2\sigma^{2}}\right)$$

Hence the information entropy of ranging error can be calculated as Formula (5).

(5) $$H\left(e_{r}\right)=\int_{-\infty }^{+\infty }p\left(e_{r}\right)\text{ln}\;p\left(e_{r}\right)de_{r}=\frac{1}{2}\text{ln}\left[2e\pi \sigma^{2}\right]$$

Let Formula (3) equal to Formula (5), the entropy uncertainty of Gaussian distribution $N\left(0,\sigma^{2}\right)$ can be derived as Formula (6).

(6) $$U=\frac{\sqrt{2\pi e}}{2}\sigma =2.07\sigma$$

The entropy coefficient of Gaussian distribution is 2.07. In general, let U=kσ, where k is called entropy coefficient and σ is the standard error deviation. The value of *k* depends on the error distribution. In this paper, the distribution of ranging error is unknown, so that *k* cannot be calculated directly. Definition 1 illustrates how to choose the value of *k*.

**Definition** **1.**  *Based on the maximum entropy principle [26] and the obtained partial information of the unknown distribution, the distribution with the maximum entropy should be selected. In all the distributions, Gaussian distribution has the maximum information entropy. Thus, the entropy coefficient of Gaussian distribution can be used to calculate the entropy uncertainty of ranging error in this paper. It is a relatively conservative but reasonable choice*.

**Proof** **of** **Definition** **1.**  Given the probability distribution of p(x) and q(x), the in Equation (7) can be obtained by using the inequation of log x≤(x−1),

(7) $$\int p\left(x\right)\text{log}\frac{q\left(x\right)}{p\left(x\right)}dx\leq \int p\left(x\right)\left(\frac{q\left(x\right)}{p\left(x\right)}-1\right)dx=\int q\left(x\right)dx-\int p\left(x\right)dx=0$$

Meanwhile, since $\text{log}\frac{q\left(x\right)}{p\left(x\right)}=\text{log}q\left(x\right)+\text{log}\frac{1}{p\left(x\right)}$, $\int p\left(x\right)\text{log}\frac{q\left(x\right)}{p\left(x\right)}dx$ can be changed into another format.

(8) $$\int p\left(x\right)\text{log}\frac{q\left(x\right)}{p\left(x\right)}dx=-\int p\left(x\right)\text{log}p\left(x\right)dx+\int p\left(x\right)\text{log}q\left(x\right)dx$$

Through Formulas (7) and (8), the in Equation (9) can be obtained.

(9) H(p)≤−∫p(x)log q(x)dx

The Formula (9) is a famous conclusion that entropy of a probability distribution is always less than the relative entropy in the information theory. Only when q(x)=p(x) can the equality hold in Formula (9).

Let $q\left(x\right)=N\left(u,\sigma^{2}\right)$, when p(x) is under the given condition of mean value u and variance $\sigma^{2}$, then the Formula (9) can be derived as follows.

(10) $$\begin{array}{ll} H\left(p\right) & \leq -\int p\left(x\right)\text{log}\left\{\frac{1}{\sqrt{2\pi }\sigma }e^{-\frac{{\left(x-u\right)}^{2}}{2\sigma^{2}}}\right\}dx\\ & =\int p\left(x\right)\left\{\frac{{\left(x-u\right)}^{2}}{2\sigma^{2}}+\text{log}\sqrt{2\pi }\sigma \right\}dx\\ & =\frac{1}{2\sigma^{2}}\int p\left(x\right){\left(x-u\right)}^{2}dx+\text{log}\sqrt{2\pi }\sigma \end{array}$$

Under the limit of the mean value and the variance of p(x):$\int p\left(x\right){\left(x-u\right)}^{2}dx=\sigma^{2}$, the inequation of $H\left(p\right)\leq \frac{1}{2\sigma^{2}}\sigma^{2}+\text{log}\sqrt{2\pi }\sigma =\frac{1}{2}+\text{log}\sqrt{2\pi }\sigma$ can be obtained. When $p\left(x\right)=N\left(u,\sigma^{2}\right)$, the equality of Formula (9) holds. Hence, the conclusion mentioned above in Definition 1 is verified.

Since the Gaussian distribution has the maximum information entropy, it has the maximum entropy coefficient. Choosing the entropy coefficient of Gaussian distribution is relatively conservative. However, based on the maximum entropy principle, the reasonable inference of the unknown distribution is the distribution which is most random and is in accord with the known information. Because this is the only choice which could be made impartially, and any other options mean that other constraints and assumptions would be added, which cannot be obtained based on the known information. Gaussian distribution is the most random distribution in nature, as we all know. Thus, the entropy coefficient of Gaussian distribution is a reasonable choice.

### 4.2. Foundation of the Trust Evaluation Model

After obtaining the entropy uncertainty of ranging error, the uncertain value of distance estimation can be written as $U_{d}=b+2.07\sigma$, where b is the mean of ranging error. More specifically, b is calculated as Formula (11),

(11) $$b=\frac{\sum_{u=1}^{n}\left(d_{ui}^{\prime }-\sqrt{{\left(x_{u}^{\prime }-x_{u}\right)}^{2}+{\left(y_{u}^{\prime }-y_{u}\right)}^{2}}\right)}{n}$$

where $d_{ui}^{\prime }$ is the measured distance between the anchor node and the unknown node, $\sqrt{{\left(x_{u}^{\prime }-x_{u}\right)}^{2}+{\left(y_{u}^{\prime }-y_{u}\right)}^{2}}$ is the computed distance between the anchor node and the unknown node, and n is the number of unknown nodes. Because the real distance between the unknown node and anchor node cannot be known in the localization process, the distance outlier needs to be detected by using the cooperation of the neighboring anchor nodes around the unknown node.

The difference between Euclidean distances and measured distances is $D_{ij}=\left|d_{ij}-d_{ij}^{\prime }\right|$, where $d_{ij}$ is the Euclidean distance between the anchor nodes and $d_{ij}^{\prime }$ (*i* = 1, 2, 3,…, *m*; *j* = 1, 2, 3,…, *m*; *i*≠*j*) is the measured distance between the anchor nodes. $d_{ij}$ is defined as $d_{ij}=\sqrt{{\left(x_{i}^{\prime }-x_{j}^{\prime }\right)}^{2}+{\left(y_{i}^{\prime }-y_{j}^{\prime }\right)}^{2}}$ (*i* = 1, 2, 3, …, *m*; *j* = 1, 2, 3, …, *m*; *i*≠*j*), where $X_{i}^{\prime }=\left[x_{i}^{\prime },y_{i}^{\prime }\right]$ (*i* = 1, 2, 3, …, *m*), $X_{j}^{\prime }=\left[x_{j}^{\prime },y_{j}^{\prime }\right]$ (j = 1, 2, 3, …, *m*) are the declared coordinates of anchor nodes, and m is the number of anchor nodes. Based on the fuzzy set theory and the neighboring anchor nodes, a trust evaluation model is constructed. In the model, the fuzzy membership function is shown as Formula (12).

(12) $$T_{ij}=\left\{ \begin{array}{lll} 1 & & D_{ij}\leq U_{d}\\ 0 & & D_{ij}>U_{d} \end{array}\right.$$

Define $T_{ij}$ as the trust value of anchor node $A_{i}$ from anchor node $A_{j}$. All these mutual trust values calculated by Formula (12) comprise the fuzzy relation matrix T.

(13) $$T=\left[\begin{array}{cccc} 0 & T_{12} & \cdots & T_{1m}\\ T_{21} & 0 & \cdots & T_{2m}\\ \vdots & \ddots & \ddots & \vdots \\ T_{m1} & T_{m2} & \cdots & 0 \end{array}\right]$$

Then give a weight matrix W to calculate the trust value of each anchor node.

(14) $$W=\left[\begin{array}{cccc} 0 & w_{12} & \cdots & w_{1m}\\ w_{21} & 0 & \cdots & w_{2m}\\ \vdots & \ddots & \ddots & \vdots \\ w_{m1} & w_{m2} & \cdots & 0 \end{array}\right]$$

where $w_{ij}=1/m-1$.

Through data fusion, an evaluation result vector S can be obtained $S=W\circ T=\left[\begin{array}{cccc} s_{1} & s_{2} & \cdots & s_{m} \end{array}\right]$, where $s_{i}$ is the trust value of anchor node $A_{i}$ and $s_{i}=\sum_{i\neq j,j=1}^{m}w_{ij}T_{ij}$.

**Definition** **2.** *Based on the majority principle, if the trust value of an anchor node is larger than 0.5, it can be concluded that this anchor node is trusted or normal. If the trust value of an anchor node is smaller than or equal to 0.5, it can be determined that the position of the anchor node is an outlier or the corresponding measured distance is an outlier. Discard the outliers and only use the trustable data to estimate the locations of unknown nodes*.

Since the presence of moving obstacles and other special situations could generate outliers temporarily, the corresponding trust values will decrease at the same time. Throughout the lifetime of the network, this kind of trust value is not credible. Therefore, all the trust values are not stored into the sensor nodes in this paper. In every localization process, the trust values will be recalculated.

## 5. MEF-Based Location Estimation Method

### 5.1. Formulation of the Localization Problem

Overall, the localization process against outliers consists of two steps. Firstly, in order to eliminate both kinds of outliers simultaneously, an outlier detection method is proposed based on the maximum entropy principle and fuzzy set theory. The first step of the localization process, named as the initial localization phase or the detecting phase is the foundation of the follow-up positioning process. Then, since not all the outliers can be detected in the detection process, the Maximum Entropy Function (MEF) method is utilized to tolerate the errors and calculate the optimal estimated locations of unknown nodes. Both steps of the localization algorithm are indispensable. Only by utilizing both steps can the localization accuracy be highly improved.

In a word, the detection method mentioned above should be applied to eliminate distance and anchor outliers in the initial localization phase. After the detecting phase, the unknown nodes utilize all their multihop communication anchor nodes to estimate their coordinates. Based on the Formula (1) in Section 3, the nodes localization problem is shown as Formula (15),

(15) $$\text{min}\;F\left(X_{u}\right)=\text{min}\;\sum_{i=1}^{m}\left|f_{i}\left(X_{u}\right)\right|=\text{min}\;\sum_{i=1}^{m}\left|\sqrt{{\left(x_{i}-x_{u}\right)}^{2}+{\left(y_{i}-y_{u}\right)}^{2}}-d_{i}^{\prime }\right|$$

where $X_{u}={\left[x_{u},y_{u}\right]}^{T}$ is the coordinate of the estimated unknown node $N_{u}$ in the localization, $X_{i}={\left[x_{i},y_{i}\right]}^{T}$ is the anchor node $N_{i}$’s declared position, and $d_{i}^{\prime }$ is the measured distance between the anchor node $N_{i}$ and the unknown node $N_{u}$.

From Formula (1), $f_{i}\left(X_{u}\right)$ is assumed equal to zero. Due to the presence of errors and outliers, $f_{i}\left(X_{u}\right)$ is not equal to zero actually. By obtaining the minimum sum of $f_{i}\left(X_{u}\right)$ (*i* = 1, 2, 3, …, *m*), the impact of the comprehensive error on the localization will be minimized. Therefore, the estimated coordinate of unknown node with the minimum sum of $f_{i}\left(X_{u}\right)$ (*i* = 1, 2, 3, …, *m*) can be as the optimal estimated coordinate in the localization.

Note that $F\left(X_{u}\right)$ is a non-smooth function and is difficult to be minimized from Formula (15). Therefore, the MEF method, which is the least biased estimate possibility on the given information and mainly used to solve the non-smooth minimum optimization problem [27], is used to estimate the locations of unknown nodes in this paper. Using the MEF method, $F\left(X_{u}\right)$ can be changed into the entropy function $F_{p}\left(X_{u}\right)$, which is smooth and obtained by the following formula,

(16) $$F_{p}\left(X_{u}\right)=\frac{1}{p}\sum_{i=1}^{m}\text{ln}\left[\text{exp}\left(pf_{i}\left(X_{u}\right)\right)+\text{exp}\left(-pf_{i}\left(X_{u}\right)\right)\right]$$

where p is called the maximum entropy factor.

Based on [27], the following properties of the entropy function $F_{p}\left(X_{u}\right)$ are listed as follows.

**Theorem** **1.**  *For any estimated coordinate* $X_{u}$ *of unknown node, (1) when* p→+∞*,* $F_{p}\left(X_{u}\right)\to F\left(X_{u}\right)$*; (2) For any* p*,* $F\left(X_{u}\right)\leq F_{p}\left(X_{u}\right)\leq F\left(X_{u}\right)+\left(\text{ln}m\right)/p$*.*

**Proof** **of** **Theorem** **1.** 

(1) Given $X_{u}^{\prime }\in R^{2}$, if there is a vector-valued function $V\left(X_{u}^{\prime }\right)$ with components $v_{i}\left(X_{u}^{\prime }\right)=\text{exp}\left[f_{i}\left(X_{u}^{\prime }\right)\right]$, where 1<i<m, then the *l_p_*-norm of $V\left(X_{u}^{\prime }\right)$ is.
  (17) $${\left\|V\left(X_{u}^{\prime }\right)\right\|}_{p}={\left\{\sum_{i=1}^{m}{\left[v_{i}\left(X_{u}^{\prime }\right)\right]}^{p}\right\}}^{1/p}={\left\{\sum_{i=1}^{m}\text{exp}\left[pf_{i}\left(X_{u}^{\prime }\right)\right]\right\}}^{1/p}$$
  Hence, $F_{p}\left(X_{u}^{\prime }\right)=\text{ln}{\left\|V\left(X_{u}^{\prime }\right)\right\|}_{p}$. Consequently,
  (18) $$\underset{p\to \infty }{\lim}{\left\|V\left(X_{u}^{\prime }\right)\right\|}_{p}=\underset{1\leq i\leq m}{\max}v_{i}\left(X_{u}^{\prime }\right)=\text{exp}\left[F\left(X_{u}^{\prime }\right)\right]$$
  That is $\underset{p\to \infty }{\lim}F_{p}\left(X_{u}^{\prime }\right)=F\left(X_{u}^{\prime }\right)$.
(2) With the properties of *l_p_*-norm, for $X_{u}^{\prime }\in R^{2}$, $F_{p}\left(X_{u}^{\prime }\right)$ is a monotonically decreasing function in terms of p, hence
  (19) $$\begin{array}{ll} 0\leq F_{p}\left(X_{u}^{\prime }\right)-F\left(X_{u}^{\prime }\right) & =\text{ln}{\left\|V\left(X_{u}^{\prime }\right)\right\|}_{p}-\text{ln}\left[\text{exp}F\left(X_{u}^{\prime }\right)\right]\\ & =\left(1/p\right)\text{ln}\sum_{i=1}^{m}\text{exp}\left\{p\left[f_{i}\left(X_{u}^{\prime }\right)-F\left(X_{u}^{\prime }\right)\right]\right\}\\ & \leq \left(1/p\right)\text{ln}\;m \end{array}$$

The theorem mentioned above describes the relationship between entropy function $F_{p}\left(X_{u}\right)$ and original function $F\left(X_{u}\right)$ when p changes. $F_{p}\left(X_{u}\right)$ converges to $F\left(X_{u}\right)$ point wisely on $X_{u}$, as p tends to infinity. Theoretically, under the given conditions, as long as p is sufficiently large, the error between the optimal solution of $F\left(X_{u}\right)$ and the optimal solution of $F_{p}\left(X_{u}\right)$ can be made arbitrarily small. However in terms of numeral calculations, when p is fairly large, the value of entropy function $F_{p}\left(X_{u}\right)$ is overflow. Therefore, in case of the overflow, Equation (16) is transformed into the following modus.

(20) $$F_{p}\left(X_{u}\right)=\sum_{1}^{m}\left|f_{i}\left(x_{u}\right)\right|+\frac{1}{p}\sum_{1}^{m}ln\left[1+\text{exp}\left(-2p\left|f_{i}\left(x_{u}\right)\right|\right)\right]$$

Derivation steps as follows.

(21) $$\begin{array}{ll} F_{p}\left(X_{u}\right) & =\frac{1}{p}\sum_{1}^{m}ln\left[e^{pf_{i}\left(x_{u}\right)}+e^{-pf_{i}\left(x_{u}\right)}\right]\\ & =\sum_{1}^{m}ln{\left[e^{pf_{i}\left(x_{u}\right)}+e^{-pf_{i}\left(x_{u}\right)}\right]}^{\frac{1}{p}}\\ & ={\sum_{1}^{m}ln\left[e^{p\left|f_{i}\left(x_{u}\right)\right|}\left(1+e^{-2p\left|f_{i}\left(x_{u}\right)\right|}\right)\right]}^{\frac{1}{p}}\\ & =\sum_{1}^{m}\left|f_{i}\left(x_{u}\right)\right|+\frac{1}{p}\sum_{1}^{m}ln\left[1+e^{-2p\left|f_{i}\left(x_{u}\right)\right|}\right] \end{array}$$

Hence, summarizing all results, the nodes localization problem can be described as Formula (22) when p→+∞,

(22) $$\text{min}\;F_{p}\left(X_{u}\right)=\text{min}\left\{\sum_{1}^{m}\left|f_{i}\left(x_{u}\right)\right|+\frac{1}{p}\sum_{1}^{m}\text{ln}\left[1+\text{exp}\left(-2p\left|f_{i}\left(x_{u}\right)\right|\right)\right]\right\}$$

where $X_{u}$ is the estimated unknown node coordinate in the localization, and p is called the maximum entropy factor. Through minimizing the entropy function $F_{p}\left(X_{u}\right)$, the estimated coordinate of the unknown node can be regarded as the optimal estimated coordinate in localization.

### 5.2. MEF-Based Localization Process

After removing the detected outliers, the MEF-based method, which has good error tolerance and calculation accuracy, is used to estimate the locations of unknown nodes in this paper. Meanwhile, it can also rapidly converge to the global optimal value by only iterating twice or three times. Based on the above discussion, the following definition can be concluded about the localization process:

**Definition** **3.**  *The entropy function* $F_{p}\left(X_{u}\right)$ *is the overall approximation to the localization function* $F\left(X_{u}\right)$*. In the localization systems, by minimizing the entropy function* $F_{p}\left(X_{u}\right)$ *and increasing* p*, the minimum* $F\left(X_{u}\right)$ *can be indirectly obtained under certain accuracy after several iterations. Thus the optimal estimated locations of unknown nodes are obtained*.

The detailed procedures of the MEF-based method for estimating the optimal locations of unknown nodes are presented in Table 1.

## 6. Performance Evaluation

In this section, simulation results are presented and discussed. For all of the simulations, the sensor nodes are uniformly distributed in a 150 m × 150 m square field. We assume a fixed transmission range *R* = 30 m for both anchor nodes and unknown nodes. The measured distance of the sensor nodes consists of two sections. One is the real distance between two nodes and the other is the measurement error. The measurement error obeys a Gaussian distribution with the mean of 0 and the variance of 1. Thus, the ranging error is set $e_{r}\sim N\left(0,1\right)$. The distance outliers, which are the measured distance attacked or disturbed by external factors, can be described as $d^{\prime \prime }=d^{\prime }\left(1+\alpha \right)$, where $d^{\prime }$ is the measured distance without attacks or disturbance, and α is the disturbed distance percentage. In each simulation, the sensor nodes of the network are deployed 100 times to compute the average localization accuracy. The default parameters of the simulation are shown in Table 2.

LMS [22] and BRSL [24] are used to compare with proposed localization method. They are both aimed at solving the problem of locating the unknown nodes in the presence of outliers. LMS is an outlier tolerance method and BRSL is an outlier detection and elimination method. Compared with these two different methods, the advantage of our method is revealed clearly in the simulations. The Average Localization Error (ALE) using in the experiment is calculated as Formula (23).

(23) $$ALE=\frac{\sum_{u=1}^{n}\sqrt{{\left(x_{u}^{\prime }-x_{u}\right)}^{2}+{\left(y_{u}^{\prime }-y_{u}\right)}^{2}}}{nR}$$

where n is the number of unknown nodes, R is the network communication radius.

In the initial localization phase, no matter the measured distance between an anchor node and unknown node or the declared anchor position is outlier, the detection result is the same. Hence, the scenarios in which distance outliers are only considered are simulated. In the outlier detection phase of the simulation, based on the maximum entropy principle and fuzzy set theory, if the trust value of an anchor node is smaller than or equal to 0.5, it can be determined that the corresponding measured distance is an outlier. Discard the outliers and only use the trustable data to estimate the locations of unknown nodes. If the trustable data left are not enough to estimate the locations of unknown nodes, the information of the neighboring unknown nodes which have been located are used. In this section, all simulations are executed in MATLAB.

### 6.1. Impact of the Number of Distance Outliers

**Figure 3:**
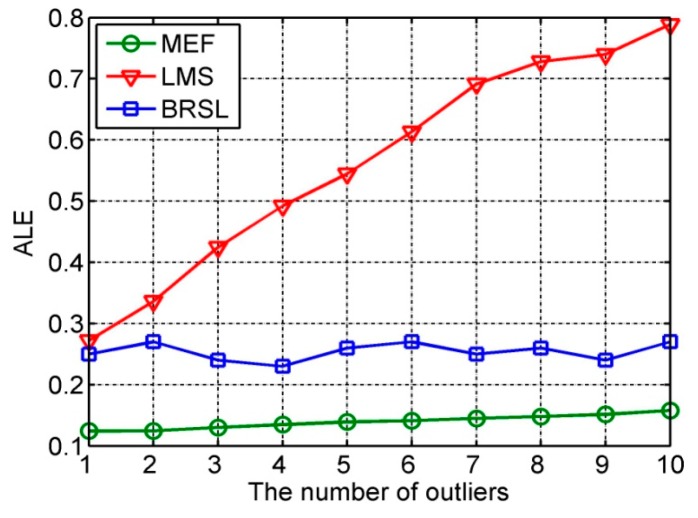
Average Localization Error (ALE) under different numbers of distance outliers.

Figure 3 shows the ALE of our localization method and the compared methods under different numbers of distance outliers. In this simulation, set α = 50% and ranging error $e_{r}\sim N\left(0,1\right)$. Simulation results show that all the detected percent of distance outliers are almost equal to 100%. With the increase of number of outliers, the ALE of LMS rises obviously, while that of MEF and BRSL remain stable, which declares that our localization is robust to the variation of distance outliers. Meanwhile, under the same numbers of distance outliers, our method can greatly improve the average localization accuracy than BRSL.

### 6.2. Impact of Disturbed Distance Percentage

**Figure 4:**
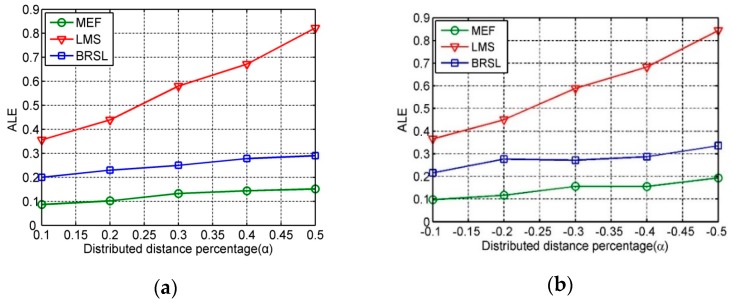
(**a**) ALE under different disturbed distance percentages when α > 0; (**b**) ALE under different disturbed distance percentages when α < 0.

Figure 4 presents the ALE of our localization method and the compared methods under different disturbed distance percentage. In this simulation, set the number of distances outliers as 10 and ranging error $e_{r}\sim N\left(0,1\right)$. In this case, the detected percent is increased to almost 100% when α=±50%. As shown in Figure 4, no matter when α>0 or α<0, the absolute difference $\left|d^{\prime \prime }-d^{\prime }\right|$ increases with the rise of |α|. In conclusion, the localization accuracy of our method decrease slowly under different disturbed distance percentages, which shows that our localization can effectively inhibit aggressive behaviors of malicious nodes and improve the localization accuracy of unknown nodes.

### 6.3. Impact of the Mean of Ranging Error

**Figure 5:**
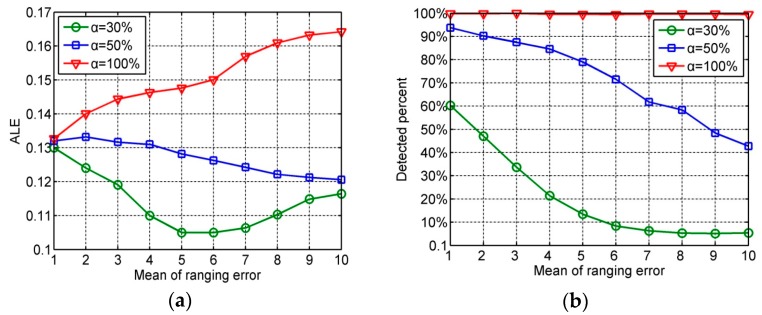
(**a**) ALE under different means of ranging errors and disturbed distance percentages; (**b**) Detected percent of distance outliers under different means of ranging errors and disturbed distance percentages.

Figure 5 illustrates the performance of our method under different means of ranging error and disturbed distance percentages. In this simulation, set the number of distances outliers as 10. Because the distance outlier detection is based on the uncertain value of distance estimation, the detected percent of distance outliers is decreased with the increasing mean of ranging error when the variation in distance is small. From the Figure 5, the localization accuracy is increased with the decreasing detected percentand the increasing mean of ranging error in the curves of α = 30% and α = 50%. Thus, it can be concluded that when the difference between the distance outlier and estimated distance is small, our localization method is error-tolerant to the undetected distance outliers; when the estimated distance is large, our localization method can detect the distance outliers.

### 6.4. Impact of the Standard Deviationof Ranging Error

**Figure 6:**
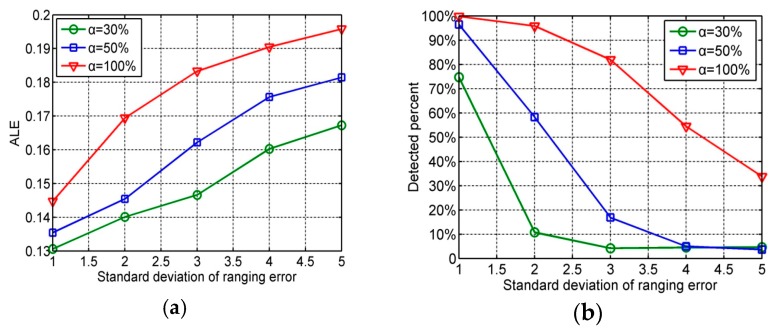
(**a**) ALE under different standard deviations of ranging error and disturbed distance percentages; (**b**) Detected percent of attacked distance estimations under different standard deviation of ranging errors and distance attacked percentages.

Figure 6 presents the performance of our localization method under different standard deviations of ranging errors and disturbed distance percentages. In the simulations, also set the number of distance outliers as 10. Compared with Figure 5, the standard deviation of ranging error has a larger impact on localization accuracy.

### 6.5. Impact of the Iteration Step Length

Note that MEF-based algorithm contains a variable parameter, i.e. the iteration step length l, the value of which will affect the performance of the algorithm. To this end, the simulation is applied to analyze the influence of step length value on algorithm performance and explain the rationality of the parameter value selection.

**Figure 7:**
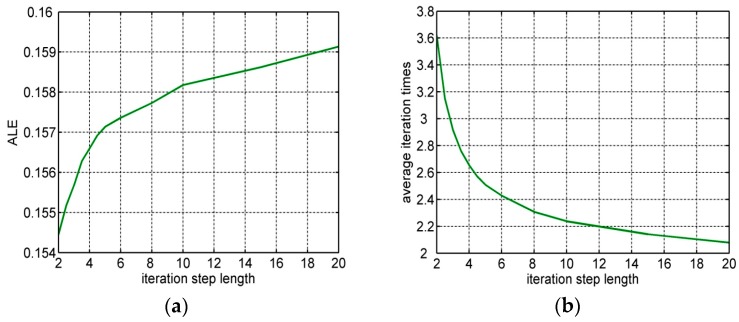
(**a**) ALE under different iteration step length; (**b**) The average iteration times under different iteration step length.

In this simulation, set the number of distances outliers to 10, p = 10, α = 50% and ranging error $e_{r}\sim N\left(0,1\right)$. Figure 7 illustrates the change trend of the average localization error and average iteration times with l increases. The increasement of iteration step length contributes to improve the efficiency of the localization. However, on the contrary, it also results in the reduction of the localization accuracy. In summary, the small value of l will reduce the efficiency of iteration. Meanwhile, the quite large value of l will decrease the localization accuracy. It should be taken into consideration that the effect of value l to the localization accuracy and the localization efficiency, when deciding the appropriate value of l in the MEF-based iteration method.

## 7. Conclusions

This paper develops an error-tolerant localization method against distance outliers and anchor outliers. First, an outlier detection method is proposed based on the maximum entropy principle and fuzzy set theory. With the cooperation of the neighboring anchor nodes of unknown node, the outliers can be detected effectively. In order to tolerate the undetected outliers and achieve high localization accuracy, MEF method is used to estimate the locations of unknown nodes. Compared with the BRSL method and LMS method, simulation results show that our localization method has higher localization accuracy.

## Figures and Tables

**Table 1:** The Maximum Entropy Function (MEF)-based method.

1: set maximum entropy factor p=10, multiple (iteration step length) *l* = 3, threshold ε = 1e-6
2: calculate the lower limit of the unknown node’s coordinate $L_{l}={\left[max\left(x_{a}^{\prime }-d_{a}^{\prime }\right),max\left(y_{a}^{\prime }-d_{a}^{\prime }\right)\right]}^{T}$
3: calculate the upper limit of the unknown node’s coordinate $L_{u}={\left[min\left(x_{a}^{\prime }+d_{a}^{\prime }\right),min\left(y_{a}^{\prime }+d_{a}^{\prime }\right)\right]}^{T}$
4: calculate the initial coordinate of unknown node $X_{u}^{\left(0\right)}=\left(L_{l}+L_{u}\right)/2$
5: **while 1**
6: minimize $F_{p}\left(X_{u}^{\left(j\right)}\right)$ and get the next iterative coordinate $X_{u}^{\left(j+1\right)}\left(j=0,1,2,\ldots \right)$
7: //determine whether $X_{u}^{\left(j+1\right)}$ is the optimal solution
8: **if** $\left|F_{p}\left(X_{u}^{\left(j+1\right)}\right)-F\left(X_{u}^{\left(j+1\right)}\right)\right|\leq \varepsilon$
9: get the optimal estimated coordinate $X_{u}^{\left(j+1\right)}$
10: **break**
11: **end if**
12: change the iterative number: j=j+1
13: change the maximum entropy factor: p=l×p
14: **end while**

**Table 2:** Default simulation parameters.

Parameters	Values
Network size	150 m × 150 m
Number of sensor nodes	150
Percent of anchor nodes	30%
Communication radius (*R*)	30 m
Hop count	2
